# A Qualitative Study Among Healthcare Providers on Risks Associated With the Use of Supportive Care for Cancer Treatment-Related Symptoms in Children and Adolescents

**DOI:** 10.1177/15347354231192959

**Published:** 2023-08-08

**Authors:** Dana C. Mora, Agnete E. Kristoffersen, Miek C. Jong, Jill A. Hervik, Trine Stub

**Affiliations:** 1 UiT The Arctic University of Norway, Tromsø, Norway; 2 Vestfold Hospital Trust, Tønsberg, Norway

**Keywords:** CAM, communication, healthcare providers, integrative medicine, pediatric oncology, qualitative, supportive care modalities, safety

## Abstract

**Introduction:**

Although more than 300 000 children and adolescents worldwide are diagnosed with cancer yearly, little research has been conducted investigating how healthcare providers consider risk and patient safety connected with supportive care (including complementary and alternative medicine [CAM]) in this age group. This study aimed to explore how different healthcare providers perceive and evaluate risk when patients combine supportive care and conventional medicine in clinical practice and how they communicate and inform parents about the use of these modalities.

**Materials and Methods:**

In-depth semi-structured interviews were conducted with 22 healthcare providers with expertise in treating pediatric oncology patients from 5 countries. Systematic content analysis was conducted using Nvivo 1.61.

**Results:**

The analysis resulted in 3 themes and 8 subthemes. Generally, participants were cautious about implementing unproven new modalities or therapies when recommending supporting care modalities to parents of children and adolescents with cancer. The most important criterion when recommending a modality was evidence for safety based on a risk/benefit evaluation. Negative interactions with conventional medicine were avoided by using the half-life of a drug approach (the time it takes for the amount of a drug’s active substance in the body to reduce by half). For patients with severe symptoms, less invasive modalities were used (ear seeds instead of ear needling). To enhance safety, participants practiced open and egalitarian communication with parents.

**Conclusion:**

Healthcare providers reported using a variety of approaches to achieve a safe practice when parents wanted to combine supportive care and conventional cancer treatment. They emphasized that these modalities should be foremost safe and not become an extra burden for the patients. Providers highlighted patient-centered care to meet the individual’s specific health needs and desired health outcomes. A lack of national and regional standardized training programs for supportive care in pediatric oncology was considered a hazard to patient safety.

## Introduction

The Supportive Care Framework for Cancer Care was originally formulated by Fitch in 1994.^1^ The framework was created as a tool for oncology experts and program managers to conceptualize what type of support cancer patients might require and how planning for service delivery might be approached. The framework draws upon the constructs of human needs, cognitive appraisal, coping, and adaptation as a basis for conceptualizing how humans experience and deal with cancer.^1^ The concepts within the framework have been validated through in-depth interviews with patients and survivors about their experiences with cancer, its treatment, and living with the aftermath of that treatment.^2,3^ Complementary and Alternative Medicine (CAM) is another tool within supportive care that aims to improve the well-being of pediatric oncology patients, and parents seek different CAM for their children as a tool to lessen the burden of cancer diagnosis and treatment.^4^

Cancer is the leading cause of death worldwide in children (0-9 years) and adolescents (10-19 years).^5,6^ The overall incidence of childhood cancer among children and adolescents in Norway is 17 per 100 000.^7^ Similar rates have been reported in Europe.^8^

The National Institutes of Health’s National Center for Complementary and Integrative Health understand complementary therapies as being nonmainstream practices applied alongside conventional medicine. In contrast, integrative medicine merges evidence-based conventional and complementary modalities in a coordinated way. The philosophical basis for many of these modalities is holistic, focusing on treating the whole person rather than a single disease or organ system.^9^ Alternative modalities refer to modalities that are used instead of conventional medicine.^9^ This practice is not supported by evidence and occurs less frequently among patients with cancer.^10^ The prevalence of the use of CAM in childhood cancer is 47% in Western countries.^11^ Parents often consider CAM modalities, such as faith healing, herbs, diet and nutrition, homeopathy and prayer, to reduce cancer treatment-related symptoms in their children.^4,12,13^

Generally, CAM is considered to be natural and, therefore, safe. However, many modalities are not independently tested by governmental agencies before being offered to the public.^14,15^ In addition, some natural products may negatively interact with cancer treatment, resulting in adverse effects and potentially negative outcomes.^16^ It is, therefore, important to investigate the possible risks of these modalities when they are used alongside conventional medicine.

Medical science risks can be divided into direct and indirect risks.^17,18^ A direct risk is due to the treatment itself. This dimension includes traditional adverse effects of an intervention, such as bleeding in response to acupuncture needling, nausea caused by chemotherapeutic medication, or an adverse effect of an herb.^19^ Indirect risk is related to adverse effects of the treatment context, for example, the CAM provider rather than the medicine. A patient can be harmed by a care context, possibly preventing the patient from receiving the best possible treatment relevant to her or his health needs.^20^ Patients often believe that the products they use are harmless or are unaware that the modality they use is considered CAM.^21^ Conventional healthcare providers do not routinely initiate open and informed discussions about the possible outcomes of combining supportive care modalities, including CAM, and conventional cancer treatment. Studies indicate that the main reason for not initiating such conversations in clinical settings is a lack of knowledge, which can create a feeling of professional discomfort.^22,23^ Discussing the use of evidence-based CAM modalities that complement conventional cancer treatment has been shown to promote its use.^24^ Primary reasons patients gave for not informing health care providers of CAM use include health care providers not asking about CAM, a feeling that health care providers were indifferent or opposed to the use of CAM, and that the use of CAM was irrelevant to their conventional cancer treatment.^25-27^

An integrative review of the information and communication needs of parents of children with cancer demonstrated that parents wanted high-quality and more reliable information about CAM from authoritative sources, primarily from conventional healthcare providers at the hospital where their child was being treated.^25^ A survey of 49 parents of pediatric cancer patients found that receiving information about CAM gave parents a sense of control and provided additional supportive treatment options.^28^ Giving parents a feeling that they were doing everything possible to support their child’s recovery. Loss of hope created despondency or desperation, and parents needed to maintain a sense of hope and control to counteract the possibility of their child’s death. The study highlighted the need for family autonomy when making CAM treatment decisions for their children.

Generally, the risk connected with the use of CAM in supportive cancer care is under-researched.^29^ With this in mind, our research team initially investigated how adverse effects were reported in the scientific literature.^30,31^ The main finding from these systematic reviews were that most of the studies included failed to report whether CAM modalities have any adverse effects. Hence it is important to investigate through research how healthcare providers handle possible adverse effects in clinical practice.

### Aim

This study was conducted as part of the research team’s efforts to develop an evidence-based decision aid for parents of children with cancer. As part of this work, we conducted this study with a twofold overall aim: (I) to explore the perceptions healthcare providers have of risk and how they evaluate patient safety when patients combine CAM and other supportive care modalities with conventional medicine in clinical practice, and (II) how they communicate and inform parents about the use of these modalities in childhood and adolescent cancer care.

## Materials and Methods

### Design

This study draws on qualitative data obtained through individual semi-structured interviews among pediatric oncology experts and CAM providers in Norway and internationally. The data obtained from the interviews were used for 2 studies. In one study we investigated the perception of supportive care use among different pediatric healthcare providers and in this present study, we investigate their perception of safety in clinical practice.^32^

Qualitative methods may contribute to a better understanding and improved level of knowledge regarding important health and well-being issues.^33^ There is a limited amount of previous knowledge regarding the combination of CAM and other supportive care modalities with conventional medicine in pediatric cancer care. It is important to understand the philosophical and medical context of these modalities.^15^ Therefore, a qualitative design is suitable for generating such information.^34^ In this study, each participant received an identification number (ID) to ensure anonymity.

### Interview Guide and Individual Semi-Structured Interviews

The developed interview guide was employed for 2 qualitative studies (see above). The interview guide was based on an integrative review of the literature and the research team’s knowledge of the literature.^25^ The interviews were semi-structured and included open-ended questions, allowing follow-up questions, and enabling the participants to give nuanced answers.^33^ The interview guide was not pilot tested and is included as supplementary material.

### Study Area and Setting

This study was initiated and designed in Norway; participants were healthcare providers from 5 countries: Canada, Germany, the Netherlands, Norway, and the United States. According to the Nordic health model all inhabitants in Norway have access to universal health care.^35^ Similar universal healthcare systems are found in Canada, Germany and the Netherlands.^36,37^ The United States has multiple healthcare systems that operate separately.^38^ CAM modalities, without regard to country, are mostly offered outside conventional healthcare systems, and patients themselves generally cover the costs of these modalities.

### Inclusion Criteria

In this study, healthcare providers were included if they: (1) had clinical experience working with CAM and/or other supportive care modalities among children and/or adolescents with cancer and (2) were trained as pediatric oncology experts (such as doctors and nurses), conventional healthcare providers (such as a physiotherapist, play-therapist, and nutritionists), or CAM providers (practicing inside or outside the conventional healthcare system at least one or more CAM modalities).

#### Participants and recruitment

Purposive and snowball sampling methods were used in this study.^39^ International healthcare providers were recruited through the research team’s professional networks. Twenty-two healthcare providers were recruited from 5 different countries (Canada [n = 1], Germany [n = 1], the Netherlands [n = 3], Norway [n = 10], and the United States [n = 7]). Five of the Norwegian participants were recruited through snowball sampling at the University Hospital of North Norway (UNN). In addition, requests were sent to CAM provider associations in Norway to identify providers with treatment experience in pediatric oncology. Five CAM providers were recruited through the Healer Association (n = 2), the Norwegian Homeopathy Association (n = 1), the Acupuncture Association (n = 1), and the Norwegian Association of Psychotherapy (n = 1).

### Data Collection

A total of 22 interviews were completed in the study. Twelve (n = 12) interviews were conducted on a web platform (Teams), enabling the participant and interviewer to see each other. Other interviews were conducted face-to-face at different workplaces (n = 9), while one interview was conducted in a private home (n = 1). The participants had no prior knowledge of the interviewer. Only the participant and the interviewer were present during the interviews. Most interviews lasted from 30 to 60 minutes. Field notes were taken by the researchers during the interviews. The last author (TS) performed the Norwegian interviews (n = 10) in Norwegian. The first author (DCM) performed the international interviews (n = 12) in English. No interviews were repeated. The last author holds a Ph.D. in medical science; she worked as a research professor when this study was carried out and is formally trained as an acupuncturist and homeopath. The first author holds a master’s in public health; she worked as a research fellow when interviews were carried out. Participants did not provide feedback on the findings of this study.

### Data Analysis

The Norwegian interviews were transcribed verbatim by a professional service. The last author (TS) translated the interviews into English. The first author (DCM) transcribed the international interviews verbatim into English. The first and last authors read them several times and created codes based on information received from each participant. Disagreements were discussed between these 2 authors until a consensus was reached. Analysis of the material was conducted according to conventional qualitative content analysis allowing the themes and codes to emerge from the data, thus inductive coding was conducted.^40^ The data was entered and coded into Nvivo 1.61.^41^ The success of content analysis depends on the coding process.

### Ethics Approval and Consent to Participate

This study is considered health service research and was registered at the Norwegian Centre for Research Data (NSD). The study was approved by NSD on 25 August 2021 (register no. 978969). Participants were informed both orally and in writing that participation in the study was voluntary. In addition, it was clear that participants could decline participation without explanation and withdraw at any time without stating a reason. Participants were further informed about the purpose and aim of the study and that data would be handled and later published and presented confidentially. Before conducting and recording the interviews, written and verbal informed consent was obtained from the participants. None of the recruited participants dropped out. The study was conducted in line with the Declaration of Helsinki and reported according to the Consolidated criteria for reporting qualitative studies (COREQ): 32-item checklist Declaration.^42,43^ (See Supplemental Material).

## Results

In this study, the themes were organized into 3 main themes (Deliberation and reflections about risk evaluation; cause no harm; cultivating patient-provider communication), and 8 subthemes (Table 1).

**Table 1.** Overview of the Main Themes and Subthemes.

Themes	Subthemes
Deliberation and reflections on risk evaluation	Safety assessment Efficacy assessment
Causing no harm	Minimizing adverse effects Minimizing Interactions Lack of standardized training
Cultivating patient-provider communication	Building trust Patient centeredness Information needs

### The Participants

Twenty-two interviews were conducted among oncology experts (n = 6), conventional healthcare (n = 4), and CAM providers (n = 12). Of these, 6 (n = 6) were trained in both conventional medicine and CAM. Participant ages ranged from 25 to 68 years (mean = 45 years). Over two-thirds of the participants were females. They were trained in 12 different supportive and CAM modalities. The majority (n = 17) had more than 10 years of experience in clinical practice (Table 2).

**Table 2.** Demographic Data of the Participants.

Healthcare providers	Total (n = 22) n (%)
Age (mean)	45.5
18-40 years of age	6 (27)
41-60 years of age	10 (45)
61 years and older	6 (27)
Gender	
Female	18 (82)
Male	4 (18)
Years in practice	
0-10 years	5 (23)
11-20 years	8 (36)
21-30 years	4 (18)
More than 31 years	5 (23)
Training*	
Oncology experts and conventional health providers	
Nurse*	5 (23)
Nutritionist	2 (9)
Pediatric oncologist*	6 (27)
Physiotherapist*	1 (5)
Play therapist	1 (5)
CAM providers	
Acupuncturist*	5(18)
Anthroposophic medicine provider*	1 (5)
Healer	3 (14)
Homeopath	1 (5)
Massage therapist	1 (5)
Music therapist	1 (5)
Psychodrama therapist*	1 (5)

* These providers were trained as both conventional and CAM providers.

### Theme I: Deliberation and Reflections on Risk Evaluation

This theme addresses how the participants deliberate about overall safety and consequences for clinical practice. The section also explores how they reflect on decision-making in their daily work.

#### Safety assessment

Safety precedence has been set by hospitals in the US offering integrative medicine. Treatments offered include energy therapies, such as touch therapies and reiki, massage, and in some instances, acupuncture—modalities that are considered safe when provided by professionals. Participant 4 emphasized that when deciding which treatments should be offered by integrative clinics, the most important factor was a proven safety record. This was confirmed by participant 7, who stated: “First and foremost, we want to make sure it [the modality] is safe before even worrying about efficacy.” The principle was confirmed by participant 22, whose philosophy was to try out modalities with evidence for safety, even though evidence for efficacy was uncertain or lacking.

Sometimes participants had difficulties accessing information about specific modalities and when that happened the modalities were routinely assessed according to a risk/benefit evaluation. This evaluation was based on information from updated research before implementation, as explained by participants 1 and 22. A pediatric oncologist stated: “[if an] integrative therapist doesn’t have information about a specific therapy, there is something called a 2 × 2 table of safety and efficacy.” If a modality was considered safe and effective (according to research literature), the modality was recommended for use. Modalities were also recommended but carefully monitored if they were considered safe even though efficacy was unknown. In situations where a modality was effective but evidence on safety was inconclusive, the modality was recommended but closely monitored for safety. Lastly, if a modality was considered not effective and connected with serious risk, it was avoided.

#### Efficacy assessment

Participants found the lack of evidence for efficacy for many CAM modalities problematic. They reflected on the consequences of their clinical practice and as a result they were conservative in terms of treatment recommendations, especially for children. This is illustrated by participant 4:

“Well, a few things, number one we know that complementary therapies . . . there is evidence supporting the use in patients in outpatient settings but there is very little [scientific, author comment] data.”

A solution to this dilemma (lack of evidence) was to suggest a substitute evidence-based modality when parents wanted to discuss a modality with a lack of evidence for an effect.

Most of the time, participants followed already established guidelines or outcomes from research published by the National Institute of Health. In addition, well-known websites/databases, such as the one from the Memorial Sloan Kettering Cancer Center were used to gather safety and efficacy information about herbs and supplements that were unfamiliar to the participants. Although there is a lack of efficacy, providers agreed with its use if the modality is safe because it contributed to the well-being of patients and their families. Participant 7 believed that “the most therapeutic part of CAM is that it gives the parent or family a sense of contribution to the process.” This sense of control was regarded as extremely therapeutic, an important element in a situation when a serious illness introduces a feeling of chaos to family life.

### Theme II: Causing No Harm

The participants emphasized the importance of preventing causing harm to patients by minimizing adverse effects and interactions of treatments. They also perceived insufficient standardized training for CAM providers as risky for patients.

#### Minimizing adverse effects

To minimize the risk of adverse effects the participants stated that treatment indication depended on the health status of the child. Participants 2 and 22 said that: “acupuncture with needles is not carried out if the patient’s absolute neutrophil count (ANC) is less than 500 cells/μL or platelets are less than 20 [20 000/μL]. These levels are set to avoid infections in the child caused by acupuncture.” Participant 2 also referred to a study conducted by their institution. She explained: “in patients with thrombocytopenia, no adverse events (including bleeding, bruising, or infections) were observed when clean needle technique protocols were employed by licensed acupuncturists who followed the safety guidelines from the National Institute of Health.”

To avoid harming children, participants assessed the health status of the child and looked at the available evidence-based data. Providers used for example ear seeds or bands instead of needles when the immune system was compromised (participant 22). Participant 23, a massage therapist, found that “patients tend to be very tired after massage.” She found reactions to massage difficult to predict and she often started with short treatments (only 10 minutes) to gauge how the body reacted.

According to participant 7, parents often asked about Reishi mushroom and there is a substantial body of research supporting its positive effects. Reishi *(Ganoderma lucidum)* is a Chinese mushroom that has demonstrated anti-inflammatory, anticancer, and anti-metastatic activities in laboratory studies. However much of the research is either based on animal models or research in adults. She (participant 7) found it challenging to discuss the uncertainty of knowing whether the mushroom would produce the same results in a 9-year-old child as it did in 400 mice (animal studies). However, she said: “What these trials have the potential to show us, is possible adverse effects which is how we can deem safety.”

To ensure documentation on safety, participant 2, a trained acupuncturist, used the hospital’s electronic medical record system to document treatment indication, frequency, and technique and to record adverse effects. The system provided access to laboratory results, including platelet and ANC count. “This documentation is in accordance with STRICTA” [standard guidelines for reporting interventions in clinical acupuncture trials, author comment] she explained.

#### Minimizing interactions

Participant 1 used the half-life of a drug method to calculate when appropriate treatment interventions could be applied in cases where parents wanted to use an herb or supplement that might negatively interact with conventional treatment. The half-life of a drug is the time it takes for the drug’s plasma concentration to be reduced to half its original value. Participant 1 explained: “a conventional drug with a 12-hour half-life (5 × 12) would no longer be present in the body after 60 hours, and at this point, the child could start taking the supplement.” This method allowed the participant to advise on when to start and stop taking the herb or supplement without affecting conventional drugs. Participants also advised parents about the advantages of using food as medicine and taking low-dose supplements. Participant 1 explained: “You can drink ginger tea, which is not going to interact with your chemo, but if you start taking 6 ginger capsules several times a day, that is not going to work with the chemo that the child is taking.”

Moreover, participant 5 explained that she will not recommend biologics (herbs) to patients who have a very good cure rate, because “I might be more nervous about offering them anything that could interfere with chemotherapy.”

#### Lack of standardized training

The major concern among the participants was the difficulty in assessing the qualifications of supportive care and CAM providers who worked outside hospitals. Different participants (2, 5, and 22) said that providers working at their respective institutions were certified professionals. “They followed evidence-based practices recommended by official entities such as the National Institutes of Health in the United States” said participant 2. However, finding a reliable CAM provider with established qualifications was difficult in most of the countries where participants were interviewed. It was especially hard in countries such as the United States and Canada, where certification requirements vary by state or province, and standardized training for CAM providers was lacking. Whether or not CAM providers had expertise in treating pediatric oncology patients was often unclear. Participant 8, who worked as an oncologist in Europe, said that “the availability of experienced complementary therapists specialized in pediatric oncology is very, very rare.”

Similarly, participant 12, a healer, believed that CAM providers need to know what to do if a patient wants to postpone or refrain from conventional treatment. “This requires training in ethics and knowledge about medical legislation,” she said (participant, 12).

According to the participants, properly trained providers decrease the possibility of putting patients at (indirect) risk, because they are trained to handle emotions and provide professional support for the child and the family to avoid medical trauma. Participant 7 believed that it is of utmost importance to have good training when working with children and cancer.

Participant 16 remembered an adolescent who became overwhelmed during a music session. Her emotions were related to her father’s despair regarding her illness. When the therapist realized that the patient could not cope with the acute situation, she terminated the therapy session carefully, postponing it to a later date when the patient was less vulnerable. Thus, she was trained to handle severe emotional traumas derived from treatment.

Participant 14, previously educated as a preschool teacher, worked as a hospital play therapist, where her objective was to try to maintain a sense of normality for hospitalized children. She was trained to teach children how to cope with difficult situations. She explained that: “hospitalized children are exposed to a lot of painful procedures. Their lives are turned upside down, routines are changed, many of them feel a loss of control, and they often become indecisive”. The department encourages role-play such as doctors and nurses. Through play, the participant observed children processing what was happening to them. She describes:

“Once I had a boy who went in and out of roles. He quoted literally everything the doctor had told him ten minutes before. The next instant, he took off his doctor’s coat and started playing with the toy train” (participant 14).

Participant 17 believed that many children with cancer try to protect their parents emotionally by pretending to be happy and smiling, even though they are crying inside. She observed parents’ suppressed emotions manifesting in children during psychodrama treatment. She remembered a girl who wanted to build houses, where each step of the process stimulated suppressed feelings of fear and sorrow. In this process, “it was important not to move forward too fast. It was all about the child being safe.” She guided the child carefully through this process based on professional training and many years of working experience.

Healthcare providers with limited training in treating children with cancer and working outside hospitals, may therefore impose a risk on these patients.

### Theme III: Cultivating Patient-Provider Communication

In the context of pediatric cancer care, communication is the key to establishing treatment goals and realistic expectations related to health care. It is, therefore, important for parents to state their needs and concerns in consultations with their medical team. This section discusses the perceptions healthcare providers have about communication through building trust, patient-centeredness, and information needs.

#### Building trust

Healthcare providers expressed that what parents felt comfortable sharing, and what they asked about, depended on the relationship they had with the healthcare providers. Participant 20, who worked as a healer, started to build trust with parents during a telephone consultation. As an experienced therapist, she knew that this initiation of contact by the parents meant that they needed to talk, so she listened. She explained: “Sometime I ended up treating both the parents and the child.”

Participants (1, 7, and 12) believed that some parents held back information about CAM use. According to the participants, the reason for nondisclosure could be that parents feared a negative response from the doctors who believed that using these modalities was a waste of money. If doctors did not include the topic of CAM modalities in conversations with patients, patients were reluctant to ask. Participant 1 believed that: “This lack of communication often leads to parents keeping quiet about treatments that had not been recommended at the hospital.”

Participant 6 appreciated that their parents seemed to trust her and were willing to have conversations about their treatment needs, including what modalities were available at the hospital and what the risks and potential benefits were. At the end of the day, there were no guarantees that families intended to follow her recommendations, but at least they had received valuable information. She said: “I would never approach (a request about CAM) with judgment; they are just trying to help their kids.” If the parents wanted to use CAM *instead of* conventional medicine for their children, participant 5 became very nervous. The reason for this was that most of the children she met had cancers that were usually cured by conventional medicine. To build trust, she was, however, “willing to go through the list of CAM modalities that could be used as a supplement to conventional medicine, together with the parents.” Moreover, the participants believed that openness was the most important factor when talking to parents. Therefore, participants encourage the parents to give them information about their use of CAM. Based on that information they could check whether the modalities were safe to use alongside conventional treatment regimes.

#### Patient centeredness

The concepts of building trust and a patient-centeredness approach complement each other. The concept of patient-centeredness was brought up by participant 8 in the interviews. He said that the lack of using this approach was problematic. He thought that doctors must be educated to train the students and the trainees in parent centeredness medicine. “That means that one of the first things I must ask is What do you think? What are your options? and What are your thoughts?.” Asking questions like that may contribute to more open and respectful patient-provider communication.

In line with patient-centered care, the play therapist’s (participant 14) main focus was always to be present for the child at that very moment. She strived to be open and receptive to what the child needed at any specific time. Working with pediatric patients meant that the participant had to be flexible and not tied to a rigid treatment regime. “Having fun was an important element,” she said.

#### Information needs

Obtaining accurate and timely information about supportive care is an important factor in enhancing safety. Getting diagnosed and starting a treatment regime is a lot to cope with for the children and their parents. Receiving treatment at the hospital was described by participants 10 as: “getting on a run-away train, moving faster and faster. After about 2 weeks, things became calmer, allowing parents time to talk and received information about supportive care modalities.”

Appropriate distribution of information to families was brought up by participants. Participant 13 believed that “a web page would be useful to relieve parents of having to seek out treatment information on their own.” She emphasized the importance of making it clear that these modalities are not a substitute for conventional treatment and are not used in a curative capacity but as complementary therapy to conventional hospital treatments. Participant 15 from Norway said: “A web page should be published nationally, rather than being attached to a specific hospital or health region. She suggested that it could be located at Helsenorge.no” [National online health service in Norway, author comment].

The participants emphasized that the most important criterion for a modality to be included in such a web page is evidence for safety. Where information about the effect, if available, should also be included. This presents a problem because scientific information is often lacking, and for some modalities, internet information is misleading according to participant 6.

Participants suggested that for the most commonly used modalities, such as acupuncture, massage, healing, and supplements, a short description including pros and cons should be included. They emphasized the importance of presenting realistic information so as not to add any extra suffering, either to the child or the family. Participant 15 said:

“I would not recommend modalities that would harm the child or that are not in the child’s best interest. If the treatment is effective and does not cause harm, I would recommend it.”

Participants suggested organizing a web page according to treatment modalities indicated for the most common symptoms associated with childhood and adolescent cancer modalities, for example, pain, obstipation, lack of appetite, and anxiety. They also pointed out the importance of including modalities that help a child cope with everyday life. Sick children still need to play, and play is an important tool that can be utilized to process emotions and painful experiences. Ways to facilitate and organize play activities were suggestions, as was practical and realistic advice about diets and nutrition. Participant 6 believed that: “Relevant advice should be tailored to different food cultures.”

Other suggestions included a list of competent CAM providers; information regarding financial support, including insurance companies or private funding; and where to find reliable information (where to go next—including a list of updated webpages).

## Discussion

To the best of our knowledge, this study is the first to report how different pediatric healthcare providers reflect on and practice patient safety about supportive modalities, including CAM. The participants were conservative when recommending these modalities to parents, meaning they were cautious about implementing unproven modalities or therapies, to prevent overtreatment and harm to patients.^44^ The participants were careful to communicate the benefits/harms of the modality for the individual.

The participants emphasized that the modalities should be foremost safe and not become an extra burden for the patients. Therefore, they recommended using less invasive modalities to treat the most vulnerable children. According to the participants, negative interactions with conventional medicine were avoided by using the half-life of a drug approach. Moreover, a lack of national and regional standardized training in pediatric oncology was perceived as a major threat to patient safety. To meet patients’ needs and to establish a trustful relationship with parents, participants reported that they practiced open and egalitarian communication to encourage parents to delineate their use of CAM modalities. Based on this information, participants could take action to avoid negative interactions with conventional treatments.

Norwegian healthcare providers expressed similar views concerning safety in a previous study examining attitudes concerning risks among complementary and conventional healthcare professionals.^45^ Seventy-four percent of the participants believed that safety was the most important criterion for recommending a CAM modality to cancer patients. Moreover, 89% of medical doctors and nurses believed that CAM modalities should be subjected to more scientific testing before being accepted by conventional healthcare providers. These findings are reflected by Maha and Shaw^46^ and Fønnebø et al.^15^ Fønnebø et al proposed a 5-phase research strategy for CAM interventions, where safety status is recommended before the assessment of efficacy. This strategy would generate evidence relevant to clinical practice and acknowledge the important but subtle differences between CAM and conventional medical practice.

Negative interactions with conventional treatment are a direct risk in cancer care.^47^ The participants reported using strategies such as the half-life of a drug approach to minimize the risk of interactions between conventional drugs and supplements. Using supplements in small doses was another strategy participants reported using with the aim to avoid interactions with conventional care treatment. According to the participants, information about these strategies was imperative for parents when planning and making decisions regarding the integration of conventional medicine and CAM, and other supportive modalities.^22^

Based on a study among pediatric oncologists, Roth et al recommended applying modalities that are considered safe in professional hands, such as music and art therapy, mindfulness, and yoga.^48^ However, severe adverse effects were reported in connection with physical activities (n = 1), yoga (n = 1), and art therapy (n = 1). A study by Goldberg et al reported anxiety, traumatic re-experiencing, and emotional sensitivity following meditation.^49^ Similar findings were reported by a participant in this study when a teenager was overwhelmed by emotions during music therapy. Professionally trained providers need skills to manage and guide patients in emotional situations and help them process emotions that arise during treatments.^50^ This is especially true in pediatric oncology where children and adolescents are vulnerable, and where supportive care modalities should not add extra burden to their suffering.^50^

The participants expressed difficulty assessing the qualifications of supportive care and CAM providers outside hospitals to refer patients. Currently, there are no standard training requirements for CAM providers working in cancer care and other healthcare settings in the EU.^51^ Mackereth et al surveyed CAM providers working in cancer care. The authors highlighted the need for training standardization for providers, where specific training regarding safe practice was considered essential.^52^ A study from Switzerland confirmed increasing interest in integrative medicine among pediatricians, supporting the need for pre-and postgraduate pediatric training related to CAM and integrative oncology.^53^ Pediatric healthcare professionals are trained to guide children through difficult treatment processes and handle emotions that arise. Healthcare providers without training may impose an indirect risk on children and their families. In Norway, there is a voluntary register for CAM providers who are members of a professional organization.^54^ The register aims to increase patient safety and consumer rights for patients seeking CAM providers.^55^

Cultivating provider-patient communication is the key to establishing patients’ treatment goals and realistic expectations of health care. To establish fruitful relationships with patients, communication needs to be transparent and open. Patient-centeredness is a concept that facilitates a more egalitarian relationship between patients and their healthcare providers.^23,56,57^ Participants suggested training doctors in this concept, to form a partnership with their patients. Facilitating equality is a prerequisite for good and effective communication.^58^ Without this joint establishment of meaning, patients are at increased risk of adverse effects and harm during medical care. Accordingly, Frenkel et al and others believe that an open and equal dialog may decrease risks associated with malpractice, maximize positive treatment outcomes, and avoid adverse effects that may occur when combining conventional treatment and supportive care.^23,56,57^

A review from 2020 concluded that there is a need for information about supportive care among parents of children with cancer.^25^ According to relevant literature parents want information from authoritative sources such as oncologist experts at hospitals.^12^ However, information sources most often consulted are family and friends and the media.^25,59,60^ Ndao et al found that where an integrated program existed, more than half of the participants would use them.^61^ In this study, providers agreed that it is important to provide practical, realistic, and easy-to-implement information, with no extra burden on the suffering of the children.

## Limitations and Strengths

This study should be interpreted in light of its limitations. The study is based on data from a selected group of healthcare providers. They were recruited through the network of the research team. Therefore, the present findings are not representative of all healthcare providers working with supportive care and CAM for pediatric cancer patients. The qualitative analysis provides insight into how participants understand and interpret situations, but it cannot be used to establish associations. However, in-depth interviews facilitated abundant material. Moreover, the participants interviewed here showed striking similarities in their clinical experience, modalities, and concerns for their patients. Saturation was reached after 20 interviews as no new information was obtained. Another strength of this study is that the interviewed healthcare providers were from 5 different countries, distributed on 2 different continents. Although healthcare providers from different countries were interviewed, no substantial differences were found in the ways safety is assessed or in the way information should be communicated to parents. The lack of substantial differences might be because childhood cancer is a rare disease, and in high-income countries, treatment from front-line clinical research has been readily incorporated into care resulting in successful treatment protocols and high-survival rates.^62^

## Implications for Practice and Research

The findings of this study have significant implications for practice and research. In practice, our findings on safety can be used to develop information tools for patients and providers that will facilitate their decision-making process. This strategy will support open communication and enhance trust among patients and healthcare providers. Networks of supportive care providers can be developed and maintained at regional and national levels. Such networks can provide reliable information on supportive care which can be exchanged. This network can also develop a list of properly trained CAM providers with experience in treating children with cancer. These strategies may increase patient safety including direct and indirect risks associated with these practices. Furthermore, as demonstrated in this study more standardized training programs are needed for providers who work and are motivated to work in this field.

The results of this study, have important implications for research. The lack of safety and efficacy information may be due to a true lack of safety data, or lack of awareness of existing data. These differences may require different interventions such as data being developed, or training/data dissemination. More importantly, it highlights the need for funding sources to conduct further research.

## Conclusions

The participants reported using a variety of approaches to safeguard their clinical practice. However, there is a lack of evidence for the effect of many supportive care modalities in pediatric oncology, which is considered a direct risk. Moreover, there is a lack of CAM providers trained in pediatric oncology, an indirect risk. Both risks are considered a hazard to patient safety. Furthermore, participants agreed that it is important to have communication where trust is the main pillar of the provider-patient relationship to improve patient care but also to shield patients from using modalities that might not be safe.

## Supplemental Material

sj-docx-1-ict-10.1177_15347354231192959 – Supplemental material for A Qualitative Study Among Healthcare Providers on Risks Associated With the Use of Supportive Care for Cancer Treatment-Related Symptoms in Children and Adolescents Click here for additional data file. Supplemental material, sj-docx-1-ict-10.1177_15347354231192959 for A Qualitative Study Among Healthcare Providers on Risks Associated With the Use of Supportive Care for Cancer Treatment-Related Symptoms in Children and Adolescents by Dana C. Mora, Agnete E. Kristoffersen, Miek C. Jong, Jill A. Hervik and Trine Stub in Integrative Cancer Therapies

sj-docx-2-ict-10.1177_15347354231192959 – Supplemental material for A Qualitative Study Among Healthcare Providers on Risks Associated With the Use of Supportive Care for Cancer Treatment-Related Symptoms in Children and Adolescents Click here for additional data file. Supplemental material, sj-docx-2-ict-10.1177_15347354231192959 for A Qualitative Study Among Healthcare Providers on Risks Associated With the Use of Supportive Care for Cancer Treatment-Related Symptoms in Children and Adolescents by Dana C. Mora, Agnete E. Kristoffersen, Miek C. Jong, Jill A. Hervik and Trine Stub in Integrative Cancer Therapies

sj-xlsx-3-ict-10.1177_15347354231192959 – Supplemental material for A Qualitative Study Among Healthcare Providers on Risks Associated With the Use of Supportive Care for Cancer Treatment-Related Symptoms in Children and Adolescents Click here for additional data file. Supplemental material, sj-xlsx-3-ict-10.1177_15347354231192959 for A Qualitative Study Among Healthcare Providers on Risks Associated With the Use of Supportive Care for Cancer Treatment-Related Symptoms in Children and Adolescents by Dana C. Mora, Agnete E. Kristoffersen, Miek C. Jong, Jill A. Hervik and Trine Stub in Integrative Cancer Therapies
