# Supplementary figures and images for: A Qualitative Study Among Healthcare Providers on Risks Associated With the Use of Supportive Care for Cancer Treatment-Related Symptoms in Children and Adolescents
Source: Integr Cancer Ther. 2023 Aug 8;22:15347354231192959. doi: 10.1177/15347354231192959 (PMC10411284; doi:10.1177/15347354231192959)

**
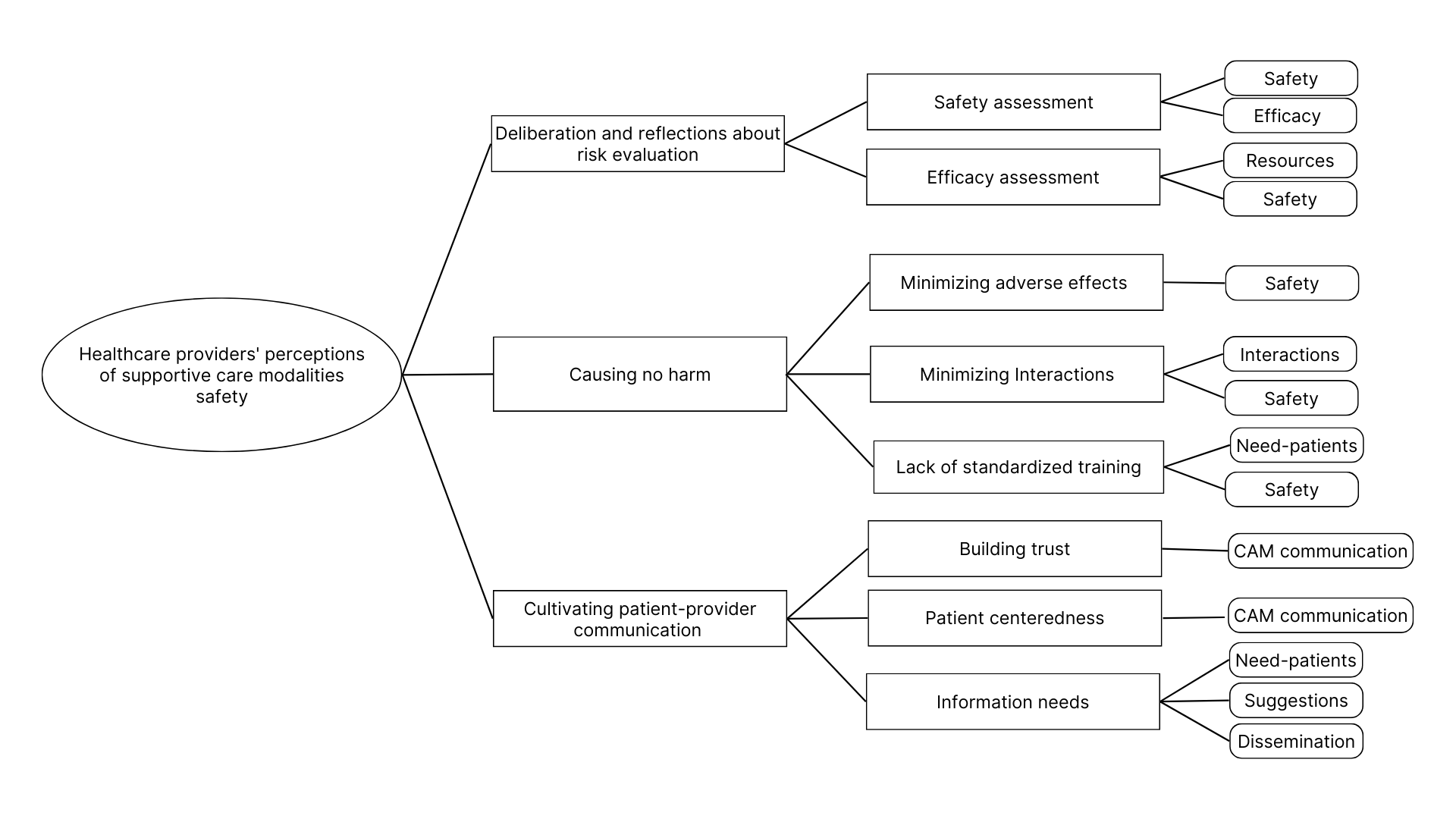
Figure S1.** Coding Tree

Supplement: sj-docx-1-ict-10.1177_15347354231192959 – Supplemental material for A Qualitative Study Among Healthcare Providers on Risks Associated With the Use of Supportive Care for Cancer Treatment-Related Symptoms in Children and Adolescents [file sj-docx-1-ict-10.1177_15347354231192959.docx]
