# Supplementary material for: A Qualitative Study Among Healthcare Providers on Risks Associated With the Use of Supportive Care for Cancer Treatment-Related Symptoms in Children and Adolescents
Source: Integr Cancer Ther. 2023 Aug 8;22:15347354231192959. doi: 10.1177/15347354231192959 (PMC10411284; doi:10.1177/15347354231192959)
Supplement: sj-docx-2-ict-10.1177_15347354231192959 – Supplemental material for A Qualitative Study Among Healthcare Providers on Risks Associated With the Use of Supportive Care for Cancer Treatment-Related Symptoms in Children and Adolescents [file sj-docx-2-ict-10.1177_15347354231192959.docx]

**Children with cancer and self-management strategies: Providers Interview Guide**

**Introduction**

- Can you please tell me about yourself and your professional background?
  - What is your profession?
  - How long have you been working in your field?
  - How long have you been working with pediatric cancer patients?

**Cancer**

- What type of cancer would you say you most often treat?
- What is the most common cancer treatment among your patients? (chemotherapy, radiation, surgery?)
- What cancer treatment induced symptom do your pediatric cancer patients most often complain about?

**Self-management strategies (SMS) and CAM**

- Do you recommend SMS/CAM to your patients?
- How long have you been working with the modality?
- When do you recommend the modality?
- Why do you recommend SMS/CAM (your philosophy)
- What are the reasons parents inquiring about this modality?
- What modalities have you used/recommended to your pediatric cancer patients? Acupuncture/Acupressure? Hypnosis? Supplements? Art music therapy? physiotherapy
- For what group of children do you recommend this treatment for?
- For what symptoms/diagnoses do you recommend this modality for? (CINV, Mucositis, Pain? Health related quality of life? Mental health?
- At what stage did you recommend/use this modality?
- What are the disadvantages of the modality?
- What obstacles do your patients have accessing the treatments?

**Safety and efficacy**

- Do you think the modality is safe? Risks?
- Did your patient have any adverse effects from the strategy?
- Do you know about interaction of conventional medicine with products? (herbs, vitamin/supplement)
- Doctor delay contact- do you have experience with this
- Experience with children/parents who decline conventional medicine/ guidelines to follow in this case
- Did the strategy have any beneficial effects?
- Do you think SMS/CAM is effective?
- Can you tell me about the efficacy and safety of modalities your patients have used but which you did not necessarily recommend?
- Can you tell me about the most promising clinical practice case concerning cancer-treatment and SMS/CAM?
- Where do you get information about these modalities? Scientific literature? Self-experience? Colleagues?

**Communication**

- Do you ask your patients about SMS/CAM strategies? If they use it?
- Do you feel you have enough knowledge about these strategies to recommend or discuss this with parents?
- Is this knowledge lacking form your medical education?
- What would you like to learn about CAM?
- How/where did you learn about SMS/CAM?
- Where do you gather scientific information about these modalities?
- What information do you need?
- Apart from the modalities we have already discuss are there other CAM or SMS you often recommend to your patients?
- Do you have any communication with other health care providers regarding your patient?
  - If communication with other health care provider exist, how is this communication?

**Information**

- What is the best way to provide information to parents of pediatric patients that interested in SMS/CAM?
- Do you present these modalities as a complementary treatment, or do you present these treatment possibilities once the parents inquire about them?
- What do you feel is the biggest obstacle for those parents that are interested in learning more about SMS/CAM in accessing further information and possible treatments?
- Do you feel your patients are comfortable addressing CAM information/treatments with you?
- What information should be included on a web page for SMS/CAM in pediatric oncology?

**CAM Providers**

- How often do you treat children with cancer?
- Their treatment goals, what modalities they recommend?
- What products do you prescribe?
- How do these products interact with conventional treatment?
- If they have experienced any adverse effects from the treatments
- Communication with conventional healthcare providers
